# Mechanical catalysis on the centimetre scale

**DOI:** 10.1098/rsif.2014.1271

**Published:** 2015-03-06

**Authors:** Shuhei Miyashita, Christof Audretsch, Zoltán Nagy, Rudolf M. Füchslin, Rolf Pfeifer

**Affiliations:** 1 Department of Informatics, University of Zurich, Andreasstrasse 15, 8050 Zurich, Switzerland; 2 Computer Science and Artificial Intelligence Laboratory, Massachusetts Institute of Technology, 32 Vassar St., Cambridge, MA 02139, USA; 3 Department of Bioinformatics, University of Wurzburg, Biocenter, Am Hubland, 97074 Wurzburg, Germany; 4 Department of Architecture, ETH Zurich, John-von-Neumann-Weg 9, 8093 Zurich, Switzerland; 5 Institute of Applied Mathematics and Physics, Zurich University of Applied Sciences, Technikumstrasse 9, 8400 Winterthur, Switzerland; 6 European Centre For Living Technology, S. Marco 2847, 30124 Venezia, Italy; 7 Institute of Academic Initiatives, Osaka University, 1-3 Machikaneyama, Toyonaka, Osaka, 560-8531, Japan; 8 Department of Automation, Shanghai Jiao Tong University, 800 Dong Chuan Rd, Min Hang, Shanghai 200240, China

**Keywords:** magnetic catalysis, enzyme, inhibitor, conformation change, autocatalysis, reaction phase

## Abstract

Enzymes play important roles in catalysing biochemical transaction paths, acting as logical machines through the morphology of the processes. A key challenge in elucidating the nature of these systems, and for engineering manufacturing methods inspired by biochemical reactions, is to attain a comprehensive understanding of the stereochemical ground rules of enzymatic reactions. Here, we present a model of catalysis that can be performed magnetically by centimetre-sized passive floating units. The designed system, which is equipped with permanent magnets only, passively obeys the local causalities imposed by magnetic interactions, albeit it shows a spatial behaviour and an energy profile analogous to those of biochemical enzymes. In this process, the *enzyme* units trigger physical conformation changes of the target by levelling out the magnetic potential barrier (activation potential) to a funnel type and, thus, induce cascading conformation changes of the targeted *substrate* units reacting in parallel. The *inhibitor* units, conversely, suppress such changes by increasing the potential. Because the model is purely mechanical and established on a physics basis in the absence of turbulence, each performance can be explained by the morphology of the unit, extending the definition of catalysis to systems of alternative scales.

## 1. Introduction

In the biochemical realm, enzymes (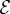) help substrates (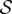) yield products (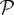) by catalysing the activation potentials of the transition paths [1]. In a typical microscopic view of catalytic reaction 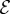 acts on 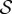, configures an enzyme–substrate complex (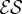), induces a conformation change of the substrate (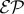) and carries off with the product, 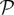
1.1
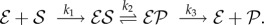
Albeit an individual molecule involves complex kinematics and is difficult to engineer, each transaction phase can be regarded as a logical operation [2], and the macroscopic view of the temporal dynamics can be characterized by the corresponding reaction speeds (*k*_1_, *k*_2_ and *k*_3_). While there is a discrepancy between the microscopic (mechanics) and the macroscopic (chemistry) perspectives, it has been generally considered that the key to this remarkable achievement lies in the addressability of individual molecules in representing discrete states, hidden in the morphology that rules the individual reaction order in a bottom-up manner.

Lately, a process (inspired by chemistry) in which components spontaneously organize into complex structures, (self-assembly), has gathered attention [3]. A typical operation is, as described in chemical engineering studies, to control a global state of a system consisting of many components by regulating an environmental agitation, inducing a composition as a product. Such a synthetic process provides a new perspective for understanding biochemical reactions, and a promising path towards new manufacturing methods for complex non-molecular composition engineering (e.g. self-assembling electronic circuits) [4]. To date, various attempts at creating artificial self-assembly systems have performed simple aggregation-based assemblies, characterized by the direct *forward reaction*. The major attempts explored in the field can be represented by a reaction equation in which the components 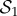 and 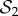 are configured into 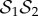, i.e. 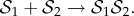 The components form a lattice structure after interacting mechanically [5], magnetically [6–10], electrostatically [11,12], via capillary forces [13–16], hydrophobic/hydrophilic forces [17,18], fluid dynamics [19] or through configuring circuitry [20–23]. Hereafter, we use the term *reaction* in a broad sense, including those obtained mechanically.

In contrast to the capability of assembly, disassembly or the so-called *backward reaction*, (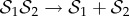), has attracted less attention, although it is critical for reconfiguration processes, catalytic reactions or regrouping components. These processes regularly combine with external forces to realize disassembly [24], or change the surrounding medium to alter the interaction between the components [25,26]. A unique approach focusing on the asymmetry of a membrane and its influence on the diffusion speeds of molecules is found in [27].

The engineering challenge here is to orchestrate an ordered assembly/disassembly down to individual components to globally attain a high yield of products, where the component has limited capabilities, as the available physical forces such as the electric, chemical or magnetic interactions provide limited interaction channels for the involved parts. Thus, for example, magnetism and capillary forces support only binary binding (either attraction or repulsion). A few notable attempts exist in which the emphases are placed on the logical responses of the components with respect to their possibilities of combining with the neighbour components, performing template replications [28–31], efficient crystallization [32] or exclusive-or (XOR) calculations [33]. These approaches actively exploit physical ‘states' of the components (e.g. 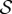 versus 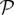), whereby the two states are realized by changes in the mechanical and/or magnetic configuration of the involved components. A state change (implicitly or explicitly) alters the terrain of the system's potential energy and, thus, has the effect of accelerating the transition from one state to another. However, little reasoning has been conducted to quantify the cause of a transition, and proposed explanations have instead been based on phenomenological descriptions with heuristically designed components. One of the reasons for this could be that the presence of environmental agitation in the system complicates these analyses (in other words, these systems are essentially open to the environment). Then, the amount of kinetic energy delivered from the environment to a component contributing to a transition over time is difficult to assess, and, thus, the component's mechanical role is difficult to evaluate in a continuous parametric space. Beyond these approaches, we expect one component to function like an enzyme, that is, to act as a third agent and enable a state switch of a targeted component (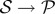) using magnetic potential energy only, where almost no environmental agitation is applied (thus, the system is essentially closed).

Here, by demonstrating that a simple enzymatic process, described by equation (1.1), can be obtained mechanically by passive magnetic units on the centimetre-scale, we focus on deriving the mechanical design principle of this chemical reaction and show that the concept of catalysis from chemistry can be generalized to alternative fields such as engineering. The proposed model, which consists of water-floating units equipped with permanent magnets, exhibits behaviours analogous to biological enzymes, and a comparable method of energy employment that levels out the potential barrier.

## 2. Design principle of magnetic catalysis

This section provides a theoretical reasoning on how the magnet motion must be coordinated in space in order to attain catalytic behaviour. We assume a physical set-up where magnets with a cylindrical shape slide on a horizontal plane, guided by physical walls.

### 2.1. Magnetism

To realize catalytic behaviour with magnets, the trajectories of the magnets must be designed at each reaction phase, which requires knowledge of the relationships between different intermagnet distances. The magnetic potential energy between two magnets *M*_1_ and *M_j_* (treated as dipoles) with magnetic moments ***m**_i_* and ***m**_j_* (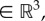 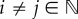) separated by a position vector *r_ij_* (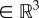) connecting their centres, is given by2.1
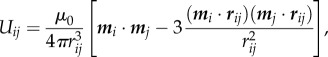
where *μ*_0_ = 4*π* × 10^−7^ H m^−1^ is the permeability of free space, and 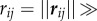 the magnet diameters.

When the magnets have an axially magnetized cylindrical shape, placed vertically on a frictionless two-dimensional plane in either parallel or anti-parallel configurations (the magnet directions are denoted by *N* or *S* in the following figures), they interact laterally. Assuming that the magnets feature the same magnetic moment magnitude 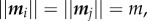 the potential and resulting forces are simplified to2.2
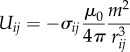
and2.3
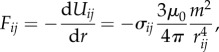
where *σ_ij_* = 1 if the magnets are anti-parallel, i.e. the two magnets are attracted along the line that connects them, and *σ_ij_* = −1 if they are parallel, i.e. repelling. We can determine the potential energy of the system, considering all involved magnets, from2.4
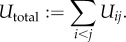
If the magnets are free to move, they will move such that the total energy is reduced (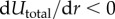), and, consequently, their relative distance is reduced (for an attractive configuration). This behaviour is the basis for designing the motion of the magnets in this work.

Equations (2.1)–(2.4) hold strictly for 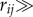 the magnet diameters, and they gradually lose accuracy as *r_ij_* becomes comparable to the diameters. However, because our model mostly needs to take the relative distances of magnets sets into account, they satisfy our requirements; in equation (2.3), the magnitudes of magnetic force strength are related to the relative distances, cancelling out the inaccuracy of their values.

**Figure 1.**
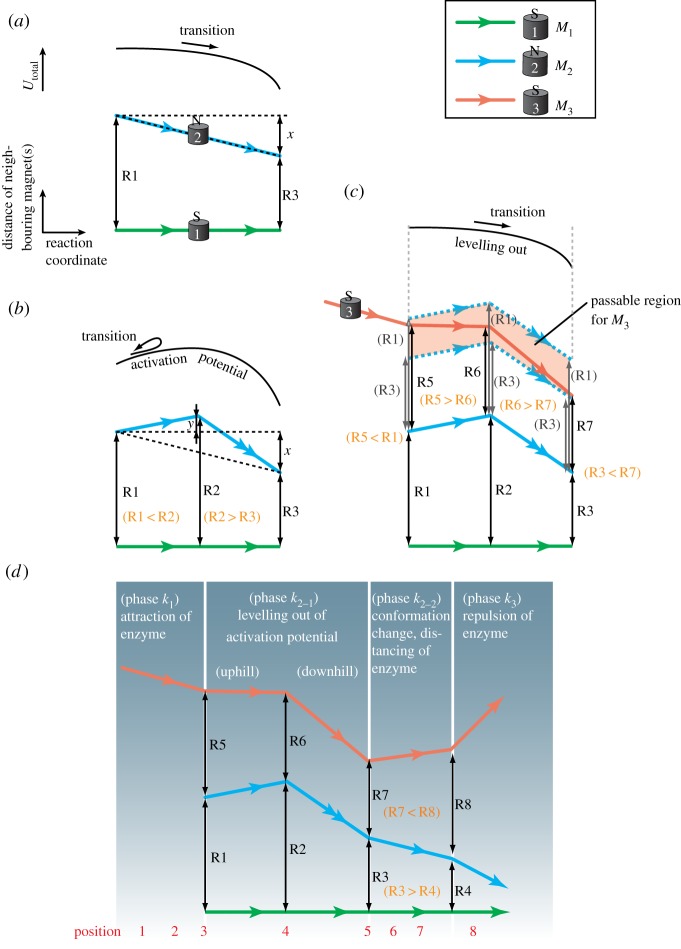
Incremental design of the enzymatic process. The lateral paths that *M*_1_, *M*_2_ and *M*_3_ follow are shown with green, blue and red lines, respectively. (*a*) Sliding motion of magnets which can potentially perform work; i.e. change the physical conformation of the units. (*b*) Activation potential. The energy profile of the outwardly wedged paths hinders the sliding motion of the magnets. The necessary conditions on the path distances are shown in orange parentheses. (*c*) Proposed magnetic catalysis. A third magnet *M*_3_ levels out the activation potential, acting as an enzyme, escorting *M*_2_ to overcome the potential barrier. The distances displayed are labelled in parentheses. The passable region for *M*_3_ derived from equation (2.5) is shown in pink. Dotted lines designate the boundaries of the path region and cannot be paths themselves. (*d*) Extension of (*c*) representing the complete enzymatic action, consisting of five distinctive phases. The position numbers 1–8, coloured in red, represent reaction stages and correspond to the same numbers in figure 2. (Online version in colour.)

The proposed magnetic catalysis is phenomenologically described in figure 1*a–d*, which provides the incremental design of the paths for the three magnets involved in the enzymatic behaviour. The horizontal dimension is the reaction coordinate, and the vertical dimension is the distance between neighbouring magnets. Note that all the magnets maintain the same horizontal coordinate positions. The paths are illustrated as straight lines for intuitive apprehension, even though the reaction speed along the horizontal axis is nonlinear. This maintains the generality of the path descriptions, because a curved function can be approximated by a combination of linear lines. We illustrate the profile of the system's potential energy *U*_total_ on top of each transition path. The state of the system is characterized by the motions of the involved magnets.

### 2.2. Sliding motion and conformation change

In figure 1*a*, if the paths of magnets *M*_1_ and *M*_2_ (anti-parallel) converge by a distance *x* (R1 > R3; *x* := R1 − R3 > 0), the magnets slide owing to the increasing magnetic attraction force, which, in turn, reduces the relative distance (R2 is not listed). The energy release can be used to perform work, which enables a kinematic reconfiguration.

### 2.3. Activation potential

The rate of physical convergence of the paths is a regulative parameter in the design. This is shown in figure 1*b*, where the choice of R1 < R2 (*y* := R2 – R1 > 0) and hence R2 > R3 create an outward wedged path acting as a ‘threshold’, which can suppress the sliding motion. Consequently, the magnets must overcome a potential energy barrier—which can be interpreted as the magnetically created activation potential—before the reaction can proceed.

### 2.4. Catalysis

Figure 1*c* illustrates the designed magnetic catalysis. The paths for *M*_1_ and *M*_2_ are the same as in the two-magnet case discussed in figure 1*b*, whereas the introduction of *M*_3_ helps the trapped magnet *M*_2_ to overcome the potential barrier and advance further on the path. When *M*_3_ is attracted towards *M*_2_, it can reach a position where the distance to *M*_2_ (R5) becomes shorter than the distance between *M*_1_ and *M*_2_ (R1). Once R1 > R5 is satisfied, and the *M*_3_ – *M*_2_ attraction exceeds that of *M*_1_ – *M*_2_, *M*_2_ begins its translation escorted by *M*_3_. Note that, owing to the quick spatial decay of the magnetic force, the net force on *M*_2_ is always dominated by the closest distance to any another magnet, and we neglect the magnetic crosstalk of the non-neighbouring magnets *M*_1_ and *M*_3_. By designing the distances in the paths as R5 > R6 and R6 > R7, we can ensure that *M*_2_ reaches an endpoint where the distance from *M*_2_ to *M*_1_ is again closer than to *M*_3_ (R3 < R7). In the end, incorporating all the distance relations above, we obtain the condition for designing paths for catalysis2.5
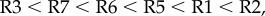
which draws the magnitude relations anticlockwise in the figure, starting from R3. Given R1, R2 and R3, we show the passable region for *M*_3_ in pink, which certifies that as long as *M*_3_ transits in this region, the reaction will proceed. The opposite happens in figure 1*b*, where the reaction stops. In this case, the system proceeds with the reaction implying that the terrain of *U*_total_ is levelled out (see the mathematical proof in appendix B). Note that the condition derived in equation (2.5) holds even if the interaction force depends on a different power of the distance, when 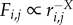 (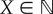).

### 2.5. Enzymatic reaction

Figure 1*d* shows the complete enzymatic reaction, which is composed of five distinctive phases. Each magnetic reaction phase can be viewed in correspondence to the three reaction phases, *k*_1_, *k*_2_ and *k*_3_, in equation (1.1). Phase *k*_1_ is when *M*_3_ is far away, approaching *M*_2_. Phase *k*_2_ is further divided into two subphases, where phase *k*_2_ *_−_*_1_ represents the event when the activation potential is levelled out, and phase *k*_2_ *_−_*_2_ represents the event when the energy is used for work, i.e. conformation change and distancing *M*_3_. From a mechanical standpoint, phase *k*_2_ *_−_*_1_ can be divided into two subphases, which correspond to the two sectors of the original activation potential, i.e. the uphill and downhill sectors, respectively. Phase *k*_2_ *_−_*_2_ is similar to phase *k*_2_ *_−_*_1_, in that all three magnets are moving, except the driving force is now between *M*_1_ and *M*_2_. *M*_1_ and *M*_2_ attract each other, decreasing the relative distance and performing the conformation change (R3 > R4). Eventually, the distance between *M*_2_ and *M*_3_ is sufficiently large to reduce the net magnetic force on *M*_3_ considerably (R7 < R8). Phase *k*_3_ is the stage when *M*_3_ is magnetically repelled. In our case, we designed the physical path of *M*_2_ such that *M*_2_ flips and changes polarity (see details in §3).

## 3. Physical substantiation

### 3.1. Units

**Figure 2.**
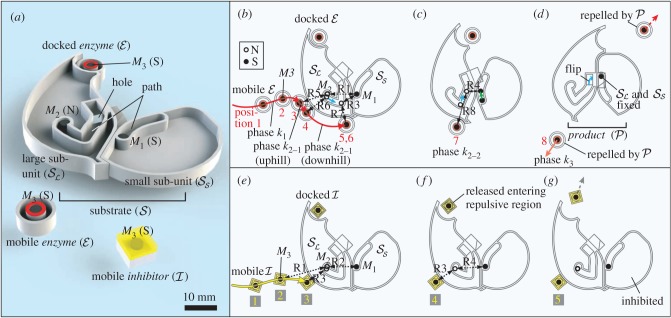
Physical model for the proposed enzymatic action, substantiated from figure 1*d*. (*a*) Overview of the designed floating units. (*b*–*d*) Magnetic catalysis by an 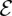 invoking a conformation change of 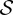, forming a different configuration 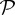; *product*. The paths of the magnets are shown in the same colours as in figure 1, with the corresponding labels R1–R8. (*e*–*g*) Inhibition of the conformation change by an 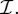 (Online version in colour.)

Figure 2*a* shows an image of the designed units that implement the three paths described in figure 1*d*. The red path represents the movement of *M*_3_ embedded in a circular unit 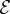 called *enzyme*. Two paths, the green path for *M*_1_ and blue for *M*_2_ are mechanically arranged and embedded in the small 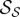 and the large 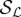 subunit as guiding walls for the magnets. Together, 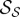 and 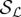 compose the *substrate* 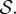 As the motion of the conformation change can be arbitrary, it is implemented such that 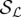 and 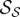, which rotate through a relative angle 90°, switch contact facets and in so doing mechanically simulate a protein's folding motion, forming a different configuration (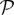; *product*). In addition, expecting to realize autocatalytic behaviour, we placed another 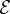 encapsulated in 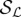 (this second 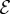 is called the docked 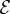 in contrast to the mobile 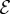). The docked 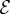 is positioned far from 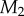, and, hence, has a small effect on the interactions of the other magnets; the existence of the docked 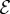 is not necessary for 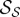 and 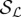 to maintain the configuration of 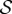.

### 3.2. Enzymatic reaction

The behaviour of an enzymatic reaction is illustrated in figure 2*b–d*, where the phases *k*_1_ –*k*_3_ comprise distinguishable stages represented by the positions of the mobile 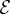 (tagged with positions red-1 to red-8). In brief, a mobile 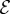 approaching from the left triggers a conformation change of 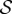, releasing the docked 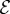, whereas it itself eventually moves away from 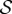 after a short contact. More concretely, the mobile 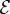 sits on the long-arc edge of 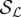, further rolls along it to a certain position where the distance between *M*_2_ and *M*_3_ (R5) becomes shorter than between *M*_1_ and *M*_2_ (R1; phase *k*_1_, positions red-1 to red-3). Note that the magnets, just as well as 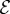, can reduce the friction with the side walls by rolling. Then, 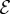 drags *M*_2_, by continuing to roll along the edge of 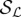 (phase *k*_2_ *_−_*_1_ uphill and downhill, positions red-3 to red-5), until the distance between *M*_1_ and *M*_2_ becomes shorter than that between *M*_2_ and *M*_3_ (R3 < R7). Then, the attraction between *M*_2_ and *M*_1_ initiates a conformation change by sliding along their respective paths, decreasing the relative distance (phase *k*_2_ *_−_*_2_, positions red-6 to red-7). Eventually, *M*_2_ falls into a hole and connects to the bottom of *M*_1_ by flipping upside-down, in the process binding the floors of 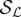 and 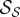. These longitudinally coupled *M*_1_ and *M*_2_ repel the mobile 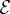 as well as the docked 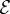 from 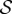 (phase *k*_3_, position red-8). When the docked 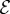 is expelled, it can subsequently act as a new mobile 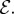 Hence, the number of mobile 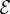 is doubled after a conformation change, inducing a cascade reaction when multiple 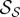 exist. The geometry R1–R8 is reflected by the paths in figure 1*d*, drawn in a proportional scale for this substantiation.

### 3.3. Inhibition

Inhibition or at least the partial suppression of a chemical reaction is also a basic biochemical function primitive, realized by highly specific molecules forming complexes with other molecules. Such molecules, called inhibitors, often dock to the binding sites of enzymes via non-covalent bonding, or prohibit conformational changes of such molecules via steric hindrance. Inspired by this fact, the inhibition of the conformation change of a *substrate* is realized by making the shape of the mobile unit rectangular, but keeping the same magnetic arrangement as for 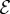 (this new unit is called an *inhibitor*, 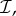 whose role is described in figure 2*e–g*). In contrast to the case of 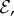 the system with 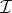 inhibits a conformation change by hindering the rotational motion of 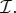 Owing to the angular shape, the mobile 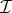 cannot roll along the edge of 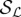 or it requires separation of two attracting magnets *M*_2_ – *M*_3_ (thus, the barrier would indeed be regarded as an activation potential by 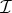), and restricts the conformation change by trapping *M*_2_ midway in its path (position yellow-4 in figure 2*f*). The docked 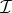 is nevertheless released because it is now in a repulsive region, and the number of mobile 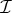 is preserved to continue reactions (see the change in the attractive region in the electronic supplementary material, figure S4). Thus, 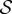 is magnetically inactivated; it cannot change its conformation nor attract another mobile 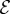 or 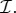 Note that reactions can proceed in parallel, because 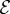 and 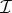 act on 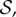 and vice versa, whereas 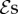 and 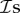 repel each other, as do the 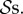


### 3.4. Experimental set-up

All the units, ranging from 7.07 to 55.66 mm in diameter (see the electronic supplementary material, figure S3 for detailed dimensions), were designed using a computer-aided design program (SolidWorks) and then printed with a three-dimensional printer (Dimension BST 768) on acrylonitrile butadiene styrene^1^. The employed magnets, *M*_1_, *M*_2_ and *M*_3_, have a cylindrical shape (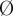 3.0 × H 3.0 mm), weigh 0.161 g and are made of nickel-coated neodymium iron boron with a magnetic flux density of 0.340 T at the middle of the surface (supermagnete, S-03-03-N). Experiments with multiple units combined in §4.2 were conducted in a water container of 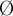
400 mm with 10 mm depth of water. Iron discs (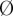
30.0 × H 3.0 mm) were placed below each 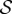 to position and maintain the initial two-dimensional coordinates of the 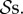 The experiments with a single conformation change were recorded by a high-speed camera, and the magnet positions were tracked using a software (Tracker).

## 4. Results

### 4.1. Conformation change and inhibition

**Figure 3.**
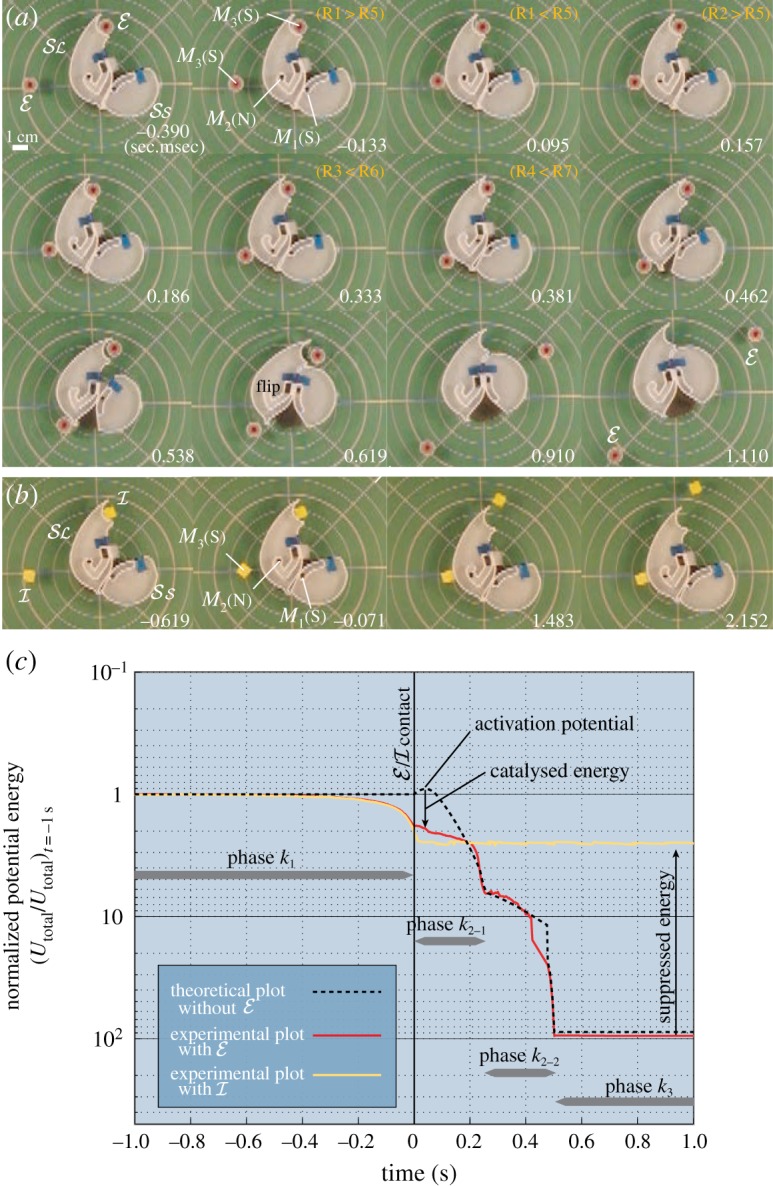
Snapshots of the conformation change by 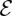 in (*a*), inhibition by 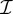 in (*b*), and the derived magnetic potential energy transitions in (*c*). (*a*) A mobile 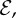 which is manually released at 8 cm to the left of the centre of the 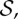 invokes a conformation change, and moves away from 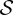 at the same time at which the docked 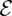 is released. (*b*) Owing to its angular shape, the mobile 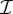 cannot roll along the long edge of 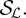 Hence, it remains at the same position, suppressing the conformation change by trapping 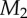 at the midpoint of its path. See the electronic supplementary material (movies S1 and S2) for these two motions. (*c*) Transitions of *U*_total_ of (*a*), (*b*), and a theoretically derived plot assuming that the conformation change occurs without 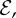 all being normalized to *U*_total_*|_t_*_=_*_−_*_1_. The vertical axis is displayed in inverted form compared with figure 1 for intuitive apprehension; the lower the point on the vertical axis the lower the energy. (Online version in colour.)

Figure 3 shows snapshots of a conformation change invoked by a mobile 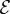 (figure 3*a*), and inhibition by a mobile 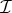 (figure 3*b*). Figure 3*a*: a mobile 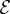 is attracted to 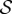 (–0.390 to –0.133 s; we set *t* = 0 s when the mobile 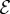 is in a contact with 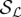), it brings *M*_2_ on 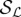 to the sharp bend in the path (0.095–0.186 s), induces a conformation change (0.333–0.619 s), and finally bears off from 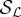 (0.910–1.110 s). The average duration of a conformation change (over 20 trials) was 0.459 ± 0.105 s (s.d.), similar to that of the contact of 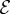 to 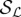 0.456 ± 0.103 s (s.d.). In most cases, at phase *k*_2_ *_−_*_1_, *M*_2_ moved faster than 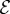, giving a brief indication of the inertia of 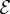.

Figure 3*b*: just as for the mobile 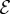 in figure 3*a*, a mobile 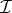, which has the same magnet arrangement, is attracted to 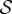 (–0.619 and –0.071 s). When 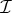 makes contact with 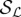, it attracts *M*_2_, but because 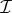 itself cannot roll along the edge of 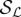, it holds *M*_2_ at the midpoint of its path, suppressing a conformation change (1.483 s). When this entrapment occurred, the docked 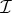 entered a repulsive region created by *M*_1_, *M*_2_ and the mobile 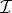, and, thus, was released from 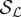 (2.152 s). This way, the released 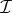 can continue the inhibition process.

Figure 3*c* displays the transitions of the system's magnetic potential energy *U*_total_, derived by analysing the spatial positions of the involved magnets (for experimental plots with 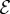 and 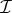), and by the design in figure 1*c* supposing that *M*_1_ – *M*_3_ transit coherently (for a theoretical plot without 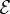). We normalized *U*_total_ by dividing by the respective *U*_total_ at *t* = –1 s (–1 s was determined arbitrarily, noticing the small magnetic influence of 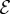 and 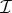). In this way, the difference in the number of magnets is cancelled out. We show the vertical axis in inverted form for an intuitive understanding corresponding to figure 1.

Owing to the catalytic effect, the potential energy of 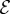 monotonically decreases, allowing the system to naturally proceed with reactions without an external energy input, and to reach a global stable state. By comparing the cases with 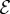 and the theoretically derived case without 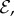 the reduction in the activation energy is clearly seen (we regard this decrease as the catalysis attained by 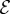). Inhibition is also clearly shown in the global stable states (e.g. *t* = 1 s), because 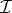 suppresses the decrease in the potential energy that 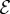 generates. The magnitude of the energy drop by 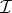 is a mere 6% of that obtained with 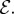 As discussed, the previously defined reaction phases can be recognized as distinctive transitions.

### 4.2. Autocatalysis with multiple units combination

**Figure 4.**
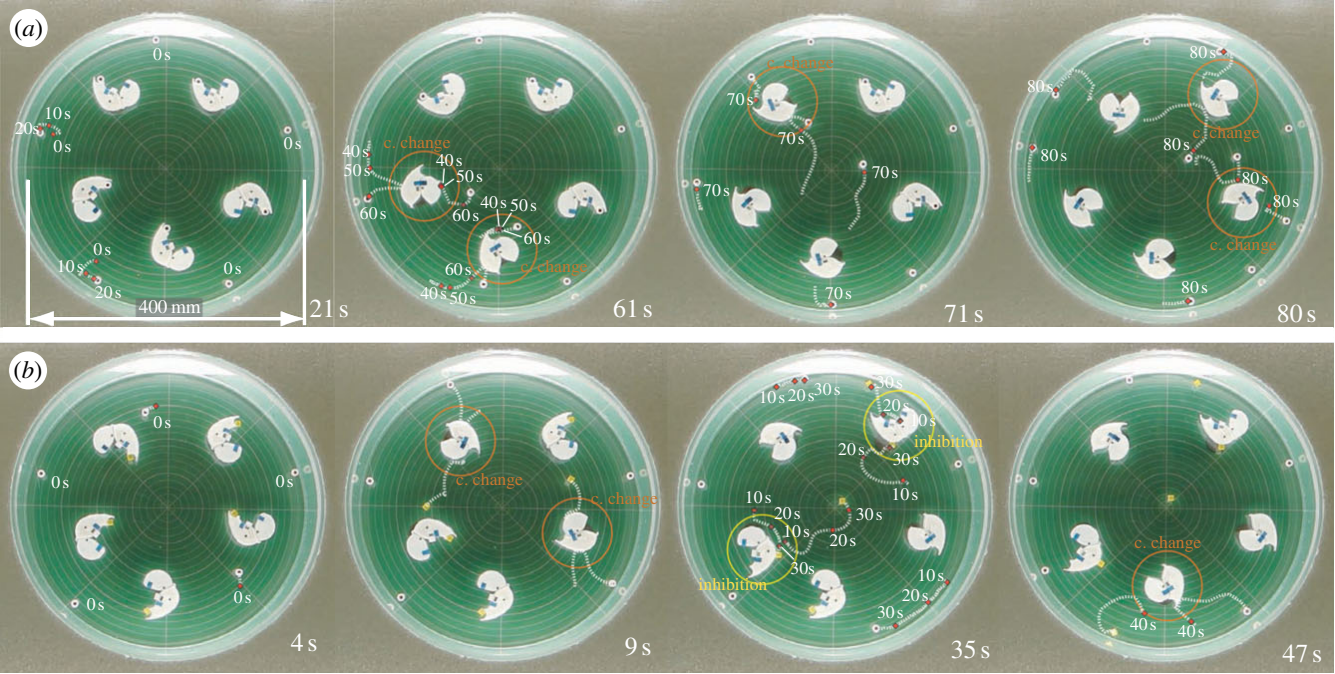
Snapshots of representative trials with a multiple unit combination for 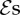 (*a*) and 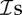 (*b*) pre-docked to the 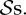 Each window displays the elapsed time after the placement of five 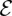 units, with trajectories of actively transitioning 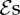 and 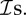 (*a*) Two of five released mobile 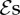 triggered conformation changes of 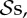 which resulted in the remaining three triggers by the recently released 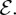 (*b*) Two mobile 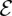 that were released by conformation changes inhibited 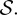 See the electronic supplementary material (movies S3 and S4) for these two cases. (Online version in colour.)

To test the designed system under more general conditions in a longer run, where multiple unit sets exist in space, we conducted experiments with five 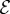
–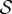 sets and 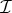
–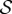 sets, and show the representative trial results in figure 4. To prevent multiple 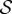 from gathering around the border of the container owing to their weak repulsion, we submerged iron plates 17 mm below the water surface level to weakly situate each 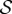, while allowing them to orient themselves in random directions. Figure 4*a* shows a trial where 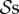 dock 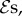 whereas figure 4*b* shows 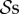 docking 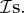 In both cases, we initiated the reactions by manually placing five 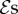 between the 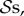 thus setting both initial conditions the same.

In figure 4*a*, after a brief interval of a quasi-stable state, the first conformation change (highlighted with a red circle) was triggered at 58.5 s, instantly followed by another conformation change at 60.1 s. The two new mobile 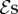 released by the conformation changes traversed the field in the 12 o'clock direction following the local magnetic field gradient, and invoked two of the remaining conformation changes (70.3 and 76.6 s). The last 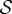 was hit by an 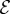 at 79.2 s, and changed its conformation. The system proceeded naturally and completed the process despite 17 of 20 magnets being involved in the series of reactions, and thus created locally complex magnetic fields. If we plot the system's energy transition as in figure 3*c*, the terrain includes sharp and significant energy drops each time a conformation change occurs. It also displays a flat terrain the majority of the time while the mobile units are travelling. The timing of the conformation changes might be affected by the density of units, though this investigation is a future research avenue.

In figure 4*b* (docked 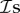), the first conformation change was triggered at 4.9 s, faster than in figure 4*a* (docked 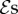). In general, the time of the first reaction is influenced by the randomly oriented 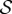, which, nonetheless, have little influence on the later reaction speed once a reaction starts. After another conformation change was triggered by an 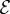 (5.9 s), the two released 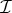 in the centre of the field inhibited different 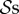 (at 9.2 and 19.6 s, highlighted with yellow circles). The final conformation change was invoked at 40.5 s. The most significant difference from the case in figure 4*a*, the docked 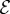 trial, is that an inhibited 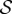 did not create as strong a repulsive magnetic field as in the case of 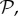 and hence it had less influence on the motion of the mobile units close by.

We conducted 30 trials for each of the two cases. The average durations for completing five reactions were 67.7 s (min = 13.3, median = 55.7, max = 146.3) with docked 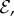 and 58.6 s (min = 11.5, median = 55.3, max = 142.7) with docked 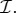 During the docked 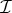 trials, there were 36 inhibitions within 150 reactions, which corresponds to 24.0% of the total number of reactions. By considering the number of invoked conformation changes, the average duration per conformation change was 13.5 s (min = 2.7, median = 11.2, max = 29.3) and 15.9 s (min = 13.3, median = 55.7, max = 146.3) with docked 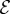 trials and docked 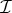 trials, respectively. The small difference in the durations is mainly because we began with 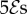 in both cases and, hence, the influence of 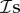 was screened.

During the experiments, a small number of trials were considered to be accidental errors, for example, because of magnets that jumped from 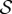 when changing conformation (this occurred five times before we reached 30 trials with 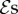), and that failed to conduct magnet flips (this occurred five times under the same conditions with 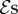). We also once terminated a trial with 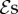 when no reaction occurred for more than 1 minute. Considering that conformation changes with a single 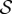 were reliable, the increase in the failure rate seems to indicate the magnetic influence of distant magnets. Within 41 potential inhibitions, 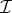 did not hold its position but slipped along the edge of 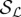 and invoked a conformation change on five occasions (failure rate 12.2%).

## 5. Discussion

Unlike highly stochastic molecular reactions where thermal agitation is the driving force for transportation and massive rapid samplings of configurations for conformation change, our system rather exhibits a deterministic behaviour, whose dynamics could thoroughly be predicted by considering the positions of all involved magnets. Our emphasis is on presenting the possibility of catalytic behaviour carried out in the almost complete absence of environmental turbulence, thus, the units' morphology with respect to each reaction phase could be discussed. This aspect of the system, that it develops rather statically, at the same time, indicates that the mechanism shows a potential for smaller scales at which the influence of mass is more negligible. Incorporation of stochasticity through externally added kinetic turbulence or water agitation could nevertheless be feasible. By sufficiently shortening R2 in figure 1*c*, but still conserving the condition that R1 < R2, such environmental perturbation may still be able to invoke a sliding motion of *M*_2_, thus realizing conformation change of 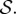 Regulating the agitation level and investigating the influence of catalytic enhancement would be of interest in future research.

## 6. Conclusion

With a special focus on the role of morphology, this study approaches the realization of a fully functional centimetre-sized, mechanical model of catalysis. We report on the construction and operation of the model, which contains both *enzymes* and *inhibitors*. To illustrate the analogous underlying processes of enzymatic behaviour, we first formulate the intermagnetic interactions attainable with permanent magnets. Then, we introduce physical units that instantiate the interaction and validate the desired behaviour where an *enzyme* triggers a kinematic reconfiguration of the target units, funnelling down the magnetic potential barrier (activation potential), whereas an *inhibitor* inhibits a reconfiguration by creating a barrier. As this phenomenon was attained at the pure physics level by combining morphology and magnetism, this study provides a platform at the intersection of classical mechanics (unit design), physics (magnetism) and chemistry (enzyme reaction). The obtained model extends the conventional definition of catalysis to systems of alternative scales, realizing ‘mechanical’ reactions with hands-on artefacts, which can expand the concept of manufacturing.

## Supplementary Material

Supplementary material of “Mechanical Catalysis on the Centimeter Scale”

## Supplementary Material

Responses to referees

## Endnote

1 The CAD data of all the designed units can be downloaded as electronic material in STL file format at http://www.shuhei.net/.
