# Supplementary material for: Mechanical catalysis on the centimetre scale
Source: J R Soc Interface. 2015 Mar 6;12(104):20141271. doi: 10.1098/rsif.2014.1271 (PMC4345491; doi:10.1098/rsif.2014.1271)
Supplement: Supplementary material of “Mechanical Catalysis on the Centimeter Scale” [file rsif20141271supp1.pdf]

Supplementary Material

Mechanical Catalysis on the Centimeter Scale

Shuhei Miyashita, Christof Audretsch, Zoltán Nagy,
Rudolf M. Füchslin, and Rolf Pfeifer

S1. Unit Outline Design

This section explains the design flow of the units described in figure S1. Once the prerequisites for the paths for the enzymatic action are determined, the design of the units is further constrained to represent the conformation change. We first designed the unit outline of $\mathcal{S}$ such that two combined units can represent a conformation change. The designed $\mathcal{S}$, ($\mathcal{S}_{\mathcal{L}}$ and $\mathcal{S}_{\mathcal{S}}$) can rotate through a relative angle of 90° (figure S1*a*), such that when two antiparallel embedded cylindrical magnets M_1 and M_2 attract and slide in the embedded paths decreasing the relative distance, the units change their contact facets, representing a conformation change (phase k_{2-2}).

Then we prolonged one of the paths on the left unit "backward" to realize phase k_{2-1}, and then phase k_1 (figure S1*b*). This process is accompanied with the determination of the edge outlines of $\mathcal{S}_{\mathcal{L}}$ and $\mathcal{S}_{\mathcal{S}}$.

Considering the formation of magnetic flux created by M_1 and M_2, which spreads symmetrically from M_1 to M_2, another $\mathcal{E}$ is embedded by extending the body of $\mathcal{S}_{\mathcal{L}}$ for autocatalytic purposes (figure S1*c*). The position was determined such that (1) it creates a reasonable size for the attractive region for a mobile $\mathcal{E}$ or $\mathcal{I}$ (ref. figure S1*a*), and (2) it is closer to $\mathcal{S}_{\mathcal{L}}$ than to $\mathcal{S}_{\mathcal{S}}$, so that the $\mathcal{E}$ is naturally attracted to $\mathcal{S}_{\mathcal{L}}$ by the magnetic force, and at the same time prevents a mobile $\mathcal{E}$ from entering the 90°–spanning region between $\mathcal{S}_{\mathcal{L}}$ and $\mathcal{S}_{\mathcal{S}}$. Note that $\mathcal{S}_{\mathcal{S}}$ and $\mathcal{S}_{\mathcal{L}}$ maintain the configuration even without a docked $\mathcal{E}$.

At the end, a bumper, a hole, and a holder were added to $\mathcal{S}_{\mathcal{L}}$ such that the bumper prevents the sliding of $\mathcal{S}_{\mathcal{L}}$ and $\mathcal{S}_{\mathcal{S}}$ while the magnets are sliding, and the hole guides the drop and flip of M_2 when a conformation change occurs (figure S1*d*). When the flip occurs, M_2 connects to the bottom of M_1 by turning upside-down, sandwiching the floors of $\mathcal{S}_{\mathcal{S}}$ and the holder of $\mathcal{S}_{\mathcal{L}}$ (flip-hold mechanism; see figure S5*a* and S5*b* for the detail flip motion).

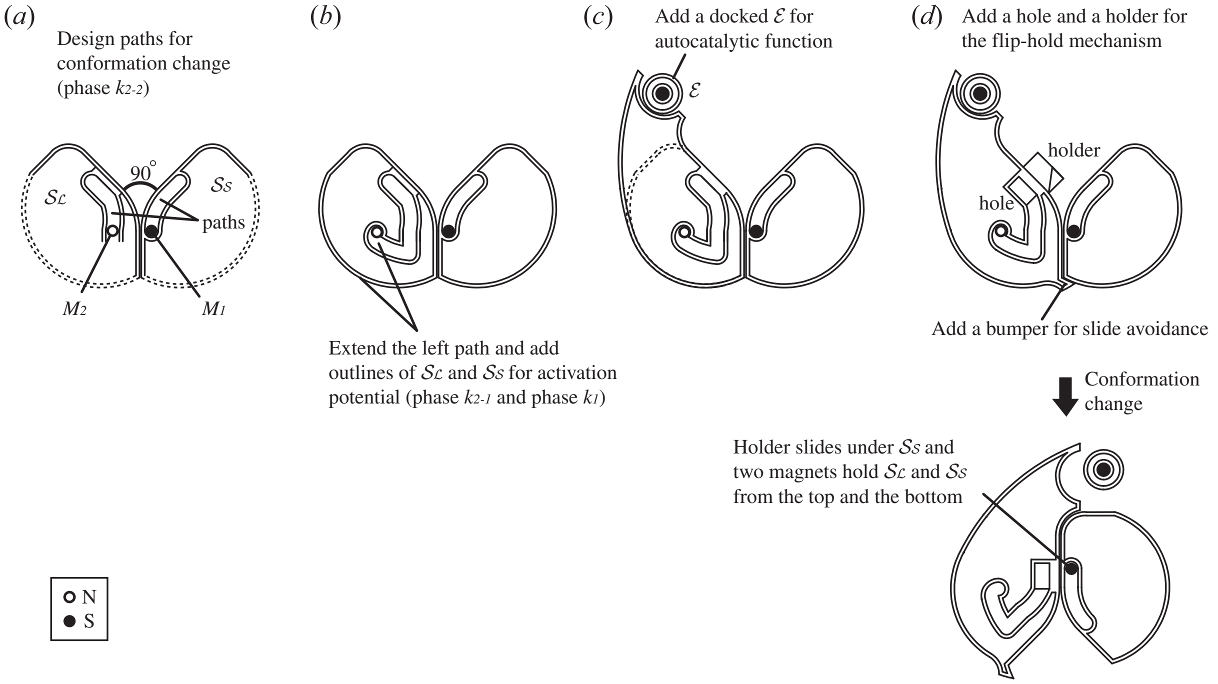

Figure S1: Unit design flow. The designs of phase k_{2-2} (*a*), phase k_{2-1}, and phase k_1 (*b*), placement of the docked $\mathcal{E}$ (*c*), and the hole and the holder (*d* top) are shown. The details are discussed in the text.

S2. Detailed Magnet Paths Implementation

This section provides supporting information to figure 1, on the most strictest condition on the path design, R5 > R6 of phase k_{2-1} (uphill), and the condition of repulsion of M_3 at phase k_{2-2}. As the colored passable region indicates, at these transitions, the sum of total relative distances could even increase (R1 + R5 < R2 + R6). We examine the accuracy of the conditions by considering the interaction of non-neighboring magnets, specifically, the interaction between M_1 and M_3. We incorporate the interaction assuming that the three magnets (M_1, M_2, and M_3) align on a straight line. Thus, the distance between M_1 and M_3 is the sum of the distance between M_1 and M_2, and between M_2 and M_3 (note that this situation rarely occurs in a physical environment). We re-illustrate figure 1*c* in figure S2 by reinterpreting the horizontal axis, which was originally a reaction coordinate, as time for an accurate physical description. The difference is that, in contrast to the reaction coordinate, time evolves at a constant rate along the axis (and cannot go backwards). We define the design parameter $\Delta_1 := \text{R1} - \text{R5}$ and the variable $\Delta_2 := \text{R5} - \text{R6}$. We also define two potentials U_3 and U_4 at the times 3 and 4, respectively, in figure S2. The goal is to find Δ_2 in relation to y such that catalysis can occur ($U_4 - U_3 < 0$) for a given Δ_1. Precisely speaking, to accurately predict the motion of the magnets, the spatial distributions of the magnetic forces need to be taken into account. However, such a calculation involves a 3-body problem, and hence requires numerical analysis. Since a general path can be broken down into linear segments, we can maintain generality and compare only the initial and final states to demonstrate the existence of the process.

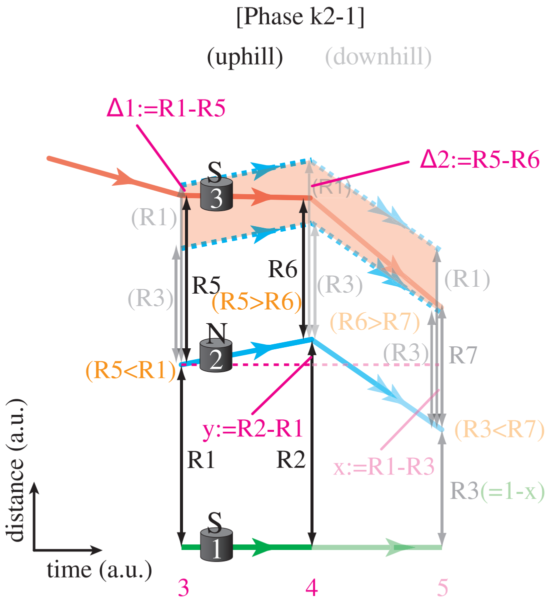

Figure S2: Re-illustration of figure 1*c* with a given design parameter Δ_1 and variable Δ_2.

We assume that R1 = 1 for simplicity, and that R5 is given (hence Δ_1 is a constant), while leaving R2 and consequently R6 as design parameters (thus y and Δ_2 are variables). The definitions lead to the following conditions; $0 < \Delta_1 < 1$, $y > 0$, $0 < \Delta_2 < 1$, and $0 < \Delta_1 + \Delta_2 < 1$.

$$U_3 = \frac{\mu_0 m^2}{4\pi}\left(-\frac{1}{R1^3} - \frac{1}{R5^3} + \frac{1}{(R1+R5)^3}\right) \quad (1)$$

$$= \frac{\mu_0 m^2}{4\pi}\left(-\frac{1}{1^3} - \frac{1}{(1-\Delta_1)^3} + \frac{1}{(2-\Delta_1)^3}\right) \quad (2)$$

$$U_4 = \frac{\mu_0 m^2}{4\pi}\left(-\frac{1}{R2^3} - \frac{1}{R6^3} + \frac{1}{(R2+R6)^3}\right) \quad (3)$$

$$= \frac{\mu_0 m^2}{4\pi}\left(-\frac{1}{(1+y)^3} - \frac{1}{(1-\Delta_1-\Delta_2)^3} + \frac{1}{(2-\Delta_1-\Delta_2+y)^3}\right). \quad (4)$$

In order for the system to proceed with the transition, $U_4 - U_3 < 0$ is required:

$$
\begin{aligned}
U_4 - U_3 < 0 \Leftrightarrow \quad & - \frac{1}{(1+y)^3} - \frac{1}{(1-\Delta_1-\Delta_2)^3} \\
& + \frac{1}{(2-\Delta_1-\Delta_2+y)^3} + 1 \\
& + \frac{1}{(1-\Delta_1)^3} - \frac{1}{(2-\Delta_1)^3} \\
(:= \ & f(\Delta_2, y)) \\
< \ & 0. \qquad (5)
\end{aligned}
$$

Theoretically, by solving equation (5), we obtain a relation between Δ_2 and y. However, while it is possible to solve the relation analytically with the help of math software, it is impractical due to the large number of expanded equation terms. Instead, we approximate f with a Taylor series to the first order in Δ_2 and y, and derive a linear relation. Let f be an infinitely differentiable function in some open neighborhood around (Δ_2, y)=(Δ_{20}, y_0). Here, we apply the Taylor expansion to f around (Δ_{20}, y_0) up to the first order (with the conditions $0 < \Delta_{20} \ll 1$, and $0 < y_0 \ll 1$), and obtain:

$$
\begin{aligned}
f(\Delta_2, y) \quad = \quad & 1 + \frac{1}{(1-\Delta_1)^3} - \frac{1}{(2-\Delta_1)^3} - \frac{3(\Delta_2-\Delta_{20})}{(-1+\Delta_1+\Delta_{20})^4} \\
& + \frac{1}{(-1+\Delta_1+\Delta_{20})^3} + \frac{3(y-y_0)}{(1+y_0)^4} - \frac{1}{(1+y_0)^3} \\
& + \frac{1}{(2-\Delta_1-\Delta_{20}+y_0)^3} + \frac{3(\Delta_2-\Delta_{20}-y+y_0)}{(-2+\Delta_1+\Delta_{20}-y_0)^4}. \qquad (6)
\end{aligned}
$$

By solving $f < 0$ while considering the domains of y and Δ_2, we obtain the condition on Δ_2 as a function of y as:

$$
\begin{aligned}
\Delta_2 \quad > \quad & \Bigg(-1 - \frac{1}{(1-\Delta_1)^3} + \frac{1}{(2-\Delta_1)^3} - \frac{3\Delta_{20}}{(-1+\Delta_1+\Delta_{20})^4} \\
& - \frac{1}{(-1+\Delta_1+\Delta_{20})^3} + \frac{3\Delta_{20}}{(-2+\Delta_1+\Delta_{20}-y_0)^4} \\
& + \frac{3y}{(-2+\Delta_1+\Delta_{20}-y_0)^4} - \frac{3y_0}{(-2+\Delta_1+\Delta_{20}-y_0)^4} \\
& - \frac{3(y-y_0)}{(1+y_0)^4} + \frac{1}{(1+y_0)^3} - \frac{1}{(2-\Delta_1-\Delta_{20}+y)^3} \Bigg) \Bigg/ \\
& \Bigg(\frac{3}{(-1+\Delta_1+\Delta_{20})^4} - \frac{3}{(-2+\Delta_1+\Delta_{20}-y_0)^4} \Bigg). \qquad (7)
\end{aligned}
$$

Substituting $y_0 = 0$ and $\Delta_{20} = 0$, we obtain

$$
\Delta_2 > \frac{(2-\Delta_1)^4 - 1}{\frac{(2-\Delta_1)^4}{(1-\Delta_1)^4} - 1} \, y. \qquad (8)
$$

By taking the limit $\Delta_1 \to 0$, indicating that M_1 and M_2 are initially equally as far apart as M_2 and M_3, we obtain

$$
\begin{aligned}
& \Delta_2 > y \\
\Leftrightarrow \quad & \mathrm{R1} + \mathrm{R5} > \mathrm{R2} + \mathrm{R6} \qquad (9)
\end{aligned}
$$

as the condition for the path design. This indicates that the sum of the relative distances between M_1 and M_2, and between M_2 and M_3 must decrease over time. For a reference, we set $R1 + R5 = 33.91\,\mathrm{mm}$ which is larger than $R6 + R2 = 32.047\,\mathrm{mm}$ in our design (note that for the majority of the time, M_1, M_2, and M_3 do not align on a straight line in our case).

By taking the limit $\Delta_1 \to 1$, i.e., that M_2 and M_3 are initially much closer than M_1 and M_2 are, which means that the influence of M_1 on M_3 is negligible, we obtain

$$
\Delta_2 > 0. \qquad (10)
$$

This condition on the original path design matches the condition R5 > R6 when the interaction of M_1 and M_3 is ignored.

The conditions of equations (9) and (10) can be applied exactly to the condition for repulsion at phase k_{2-2}, simply by swapping M_1 and M_3. On the other hand, at phase k_{2-2}, the two closely positioned magnets M_1 and M_2 now initiate the transition, distancing M_3. The possible distancing speed of M_3 is determined by the distance from M_1 and M_2 to M_3 as well as the rate of decrease of the distance between M_1 and M_2.

The substantiated paths of M_1, M_2, and M_3, described in figure 1*c* are shown in detail in figure S3*a* overlaid on an outline of the unit. The paths, which consist of segments of circles and a straight line, are decomposed into the different phases as shown in figure S3*b*-*e*. The decreases in the relative distances of the magnets were designed such that the sliding mostly occurs along the circular arcs. By shifting the relative center positions and by altering the radii of the circles, the relative distance of two magnets can be decreased gradually. Unlike the negligible friction between a magnet and the unit floor, the effect of the friction from the walls when two magnets attract cannot be ignored. The cylindrical shape of the magnets and $\mathcal{E}$ allows a rotational motion along the vertical axis, i.e., rolling, which reduces this issue.

Figure S3*b*: Without considering $\mathcal{E}$, the path functions as an activation potential as explained in figure 1*b*. At first, M_1 and M_2 are placed at positions green-3 and blue-3, respectively. Circle A has its center at green-3, and the radius is the distance to M_2 (R1). By drawing another circle (circle B) which goes through green-3, such that part of the circumference protrudes outside of circle A, a monotonic increase of the relative distance starting from green-3 to red-4 (the farthest position from green-3) is ensured (R1 < R2). By setting the path of M_2 along this segment, we expect that the motions of M_1 and M_2 are restricted.

Figure S3*c*: Circle D is set for the trace of M_3, representing phase k_{2-1}. To escort M_2 from position blue-3 to the further track, M_3 has to come closer to M_2 than to M_1 by entering the inside of circle C. This is managed by setting a trace shown as circle D, where a segment of the circumference comes inside circle C, which describes the distance to M_1 (R5 > R6). When M_3, attracted by M_2, approaches $\mathcal{S}_\mathcal{L}$, it eventually reaches red-3, travels further, and reduces the distance to M_2 by following the path of circle D (the movement from red-3 to red-4). The relative distance between M_2 and M_3 decays monotonically until M_3 reaches the point $(-24.04, -3.98)$ and M_2 the point $(-15.22, 9.25)$.

Figure S3*d*: Subsequently, M_2 enters the track of circle E, and continues the sliding accompanied by M_3 until both reach the positions of blue-5 and red-5, respectively. This is still part of phase k_{2-1}, although on the downhill side of overcoming the activation potential. During these transitions, M_1 stays at the same place (green-3, 4, 5). When M_2 and M_3 reach blue-5 and red-5, respectively, M_1 at green-5 is closer to M_2 than to M_3 (R3 < R7, ref. circle F). This now gives an initiative to M_2 to continue the motion with M_1, which brings the system to the next stage.

Figure S3*e*: When M_2 shifts toward being attracted by M_1 at green-5, and once M_2 reaches position blue-6, both M_1 and M_2 enter the tracks of circles C and G, respectively, and subsequently slide farther, reducing the relative distance (to green-7 and blue-7, and further). This induces a switch of the contact facets of $\mathcal{S}_\mathcal{L}$ and $\mathcal{S}_\mathcal{S}$ (conformation change, phase k_{2-2}). Meanwhile, M_3 is transported far from M_1 and M_2.

The next step, the flip motion of M_2, is achieved mainly by magnetic attraction to M_1. Since the orientation of M_1 is constrained to one plane, M_2, having dropped to a lower level guided by the inclined floor, automatically enters the hole and flips upside–down (see figure S5). Consequently M_1 and M_2 bind $\mathcal{S}_\mathcal{L}$ and $\mathcal{S}_\mathcal{S}$ via the bottom of $\mathcal{S}_\mathcal{S}$ and the holder of $\mathcal{S}_\mathcal{L}$, thus forming $\mathcal{P}$.

The trajectory when $\mathcal{I}$ is involved is shown in figure S3*f*. When $\mathcal{I}$ makes contact with $\mathcal{S}_\mathcal{L}$, M_2 is pulled by the M_3 on $\mathcal{I}$ (blue-4). In order for $\mathcal{I}$ to roll along the edge of $\mathcal{S}_\mathcal{L}$, the system must bring M_3 farther from M_2 than the current position (r3 < 17.10, corresponding to an activation potential for $\mathcal{I}$). Therefore without an external force, $\mathcal{I}$ cannot roll along the edge of $\mathcal{S}_\mathcal{L}$ like $\mathcal{E}$ can. Hence, this restricts the conformation change of $\mathcal{S}$.

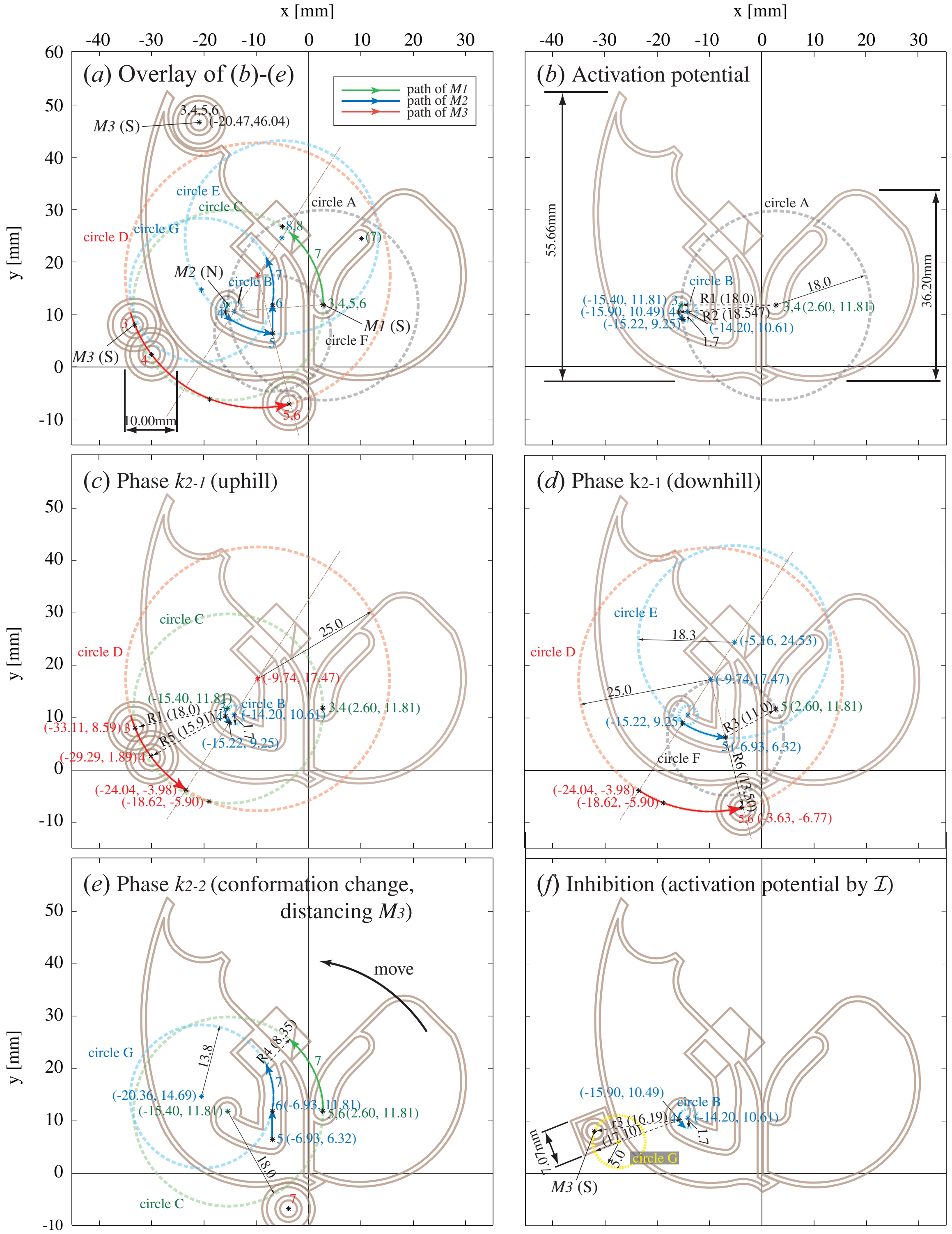

Figure S3: Implementation of the magnet paths. The paths, which consist of segments of circles, are shown with solid arrows, while the remainder of the geometry is in dashed lines for visual reference. For simplicity, we indicate a circle and its center with the same color. Labels R1-R7 represent the distances that were defined in figure 1. The tagged numbers 1-7 in green, blue, and red correspond to the same numbers in figure 2*e*-*g*, where these numbers represent the positions of each magnet at that time. (*a*) All the paths of magnets M_1, M_2, and M_3, when a conformation change occurs overlaid on the unit outline. (*b*) Activation potential. M_2, located at blue-3 is trapped by M_1 at green-3. (*c*) Phase k_{2-1}, uphill. A mobile $\mathcal{E}$ reaches $\mathcal{S}$ and rolls along the edge of $\mathcal{S}_{\mathcal{L}}$ while being attracted to M_2. (*d*) Phase k_{2-1}, downhill. The $\mathcal{E}$ and M_2 proceed with the translational motion, decreasing the relative distance, and eventually reach red-5 and blue-5, respectively. (*e*) Phase k_{2-2}. By setting the distances R3 < R6, M_1 and M_2 initiate the next step in the motion, invoking a conformation change, and at the same time moving $\mathcal{E}$ away from $\mathcal{S}$. (*f*) Inhibition. Due to the angular shape, the rectangular unit $\mathcal{I}$ cannot roll along the edge of $\mathcal{S}_{\mathcal{L}}$. Thus, it traps M_2 at the midway of the path, inhibiting the conformation change.

S3. Attractive Region

The attractive region of $\mathcal{S}$ to $\mathcal{E}$ at phase k_1 is illustrated in figure S4*a*. The figure provides a visualization of the tangential lines of the magnetic field created by M_1, M_2, and M_3 on the docked $\mathcal{E}$, as paths of mobile $\mathcal{E}$ according to equation (2.4). A mobile $\mathcal{E}$ trapped in the attractive region is pulled toward $\mathcal{S}$, whereas when it is outside this region it is expected to move away from $\mathcal{S}$. The curvature of $\mathcal{S}_{\mathcal{L}}$ is arranged with consideration of the shape of the attractive region, such that the contact can occur at any position. In practice, the interactive force induces a rotational movement of $\mathcal{S}$ and often captures an $\mathcal{E}$ near the region. When there are multiple $\mathcal{S}$s in the field, the terrain deforms depending on the positioning, angles, and the states of the neighbor $\mathcal{S}$. Since that terrain changes dynamically, a mobile $\mathcal{E}$ is delivered to an attractive region with a certain probability. Note that the shape of the attractive region for $\mathcal{I}$ is the same as $\mathcal{E}$, for the same magnetic arrangements. The formation of the magnetic field also shows that the $\mathcal{S}$s would repel each other if they were not captured by the submerged metallic plates.

Figure S4*b* shows the disappearance of the attractive region after a conformation change. The gradient of the magnetic potential energy is created jointly by M_1 and M_2, which are pointing in the same direction, exerting a strong repulsive force on $\mathcal{E}$.

After the inhibition of $\mathcal{S}$ by $\mathcal{I}$, no attractive region can be identified (figure S4*c*).

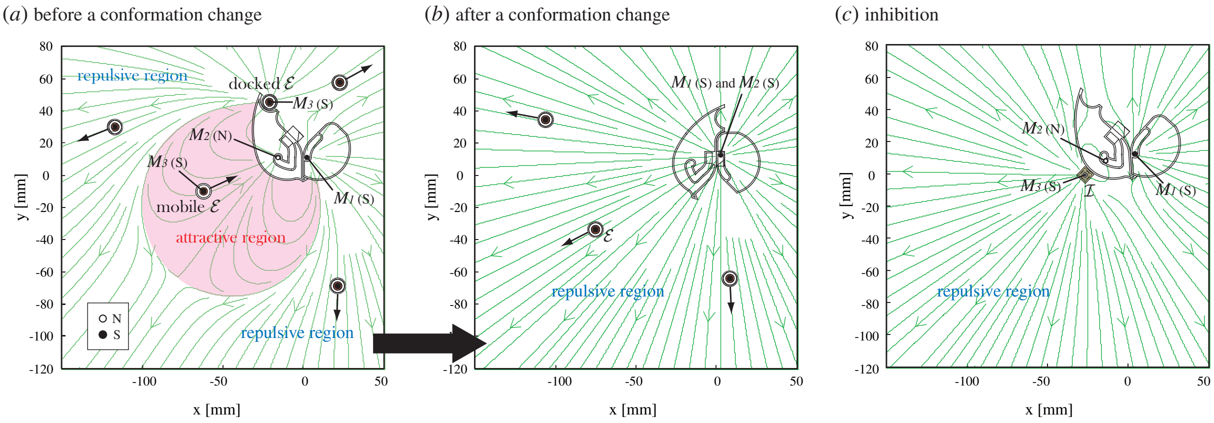

Figure S4: Paths that a mobile $\mathcal{E}$ follows, as created by the magnetic fields of M_1, M_2, and M_3 on the docked $\mathcal{E}$. (*a*) The attractive region appears next to $\mathcal{S}_{\mathcal{L}}$. (*b*) Disappearance of the attractive region after a conformation change. (*c*) Inhibition by $\mathcal{I}$, forming a magnetic field similar to the one in (*b*).

S4. Interaction of the magnets during the flip of M_2

The flip of M_2 was designed such that the magnetic potential energy U_{total} created among M_1, M_2, M_3 (on the mobile $\mathcal{E}$), and M_3 (on the docked $\mathcal{E}$) continuously decreases while M_2 is flipping and connecting to the bottom of M_3. The flip-hold mechanism is described in figure S5*a* in an angled view, and S5*b* in top view. Here, M_2 is colored red for better visualization. The positions and postures were tracked and visually estimated by using high speed video recording.

When M_2 reaches the entrance of the hole, due to the slope of the floor, the posture of M_2 inclines, thus inducing a natural flip. Once M_2 tilts, the flip motion is largely guided by the strong magnetic force of M_1. Typical positions of the magnets and the normal vectors are tagged with time labels (t0 – t4), whose corresponding times are shown in table S1. In the table, the local coordinate originates in $\mathcal{S_L}$, where $z = 0$ is set at the bottom of $\mathcal{S_L}$ (see the origin of the coordinate in the figure), and time started when a mobile $\mathcal{E}$ came in contact with $\mathcal{S_L}$. When M_1 and M_2 are combined, the center-to-center distance between the magnets is 5 mm. Given that the diameter of the magnet is 3 mm, the magnetic dipole model used to derive the magnetic potential energy in figure 3*c* is applied at the boundary conditions for this case. The inaccuracy that may stem from this would potentially appear as the last point in the energy drop plot (note that the scale is logarithmic) and, hence, has little effect on the results.

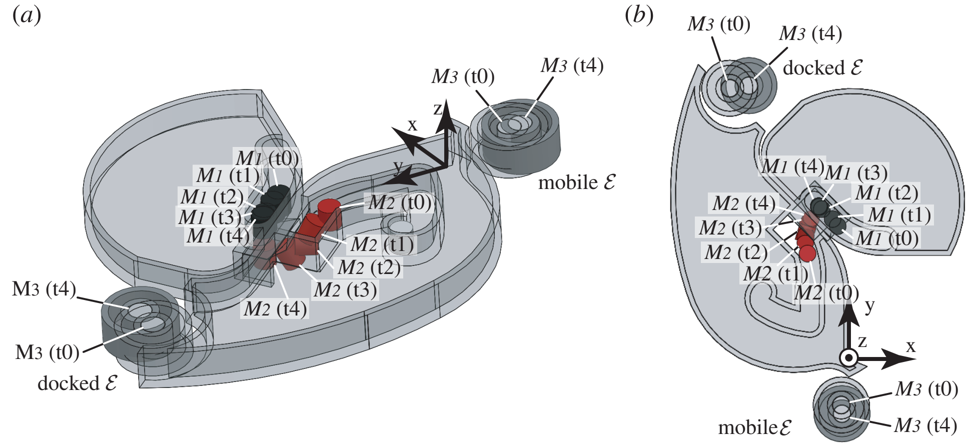

Figure S5: Flip motion of M_2 in an angled view (*a*), and top view (*b*).

Table S1: Position and normal vectors of M_1, M_2, M_3 on the mobile $\mathcal{E}$, and M_3 on the docked $\mathcal{E}$ when M_2 flips. Time is measured in ms, positions in mm.

		M_1		M_2	
time		position $[x,y,z]$	normal vector $[x,y,z]$	position $[x,y,z]$	normal vector $[x,y,z]$
t0	423.81	$[-1.97, 23.54, 2.40]$	[0.00, 0.00, 1.00]	$[-7.33, 18.65, 2.40]$	[0.00, 0.00, −1.00]
t1	478.57	$[-2.89, 24.80, 2.40]$	[0.00, 0.00, 1.00]	$[-7.69, 21.27, 1.14]$	[0.027, 0.47, −0.88]
t2	488.10	$[-4.79, 26.56, 2.40]$	[0.00, 0.00, 1.00]	$[-7.68, 22.20, -0.87]$	[0.29, 0.96, 0.057]
t3	495.24	$[-5.23, 26.98, 2.40]$	[0.00, 0.00, 1.00]	$[-7.04, 24.28, -2.37]$	[0.23 0.41 0.88]
t4	502.38	$[-5.35, 27.09, 2.40]$	[0.00, 0.00, 1.00]	$[-5.35, 27.09, -2.60]$	[0.00, 0.00, 1.00]
		M_3 (on mobile $\mathcal{E}$)		M_3 (on docked $\mathcal{E}$)	
time		position $[x,y,z]$	normal vector $[x,y,z]$	position $[x,y,z]$	normal vector $[x,y,z]$
t0	423.81	$[14.06, -20.14, 2.40]$	[0.00, 0.00, 1.00]	$[-5.77, 36.28, 2.40]$	[0.00, 0.00, 1.00]
t1	478.57	$[14.09, -20.53, 2.40]$	[0.00, 0.00, 1.00]	$[-4.92, 36.40, 2.40]$	[0.00, 0.00, 1.00]
t2	488.10	$[14.13, -20.92, 2.40]$	[0.00, 0.00, 1.00]	$[-4.07, 36.52, 2.40]$	[0.00, 0.00, 1.00]
t3	495.24	$[14.16, -21.30, 2.40]$	[0.00, 0.00, 1.00]	$[-3.21, 36.63, 2.40]$	[0.00, 0.00, 1.00]
t4	502.38	$[14.19, -21.69, 2.40]$	[0.00, 0.00, 1.00]	$[-2.36, 36.75, 2.40]$	[0.00, 0.00, 1.00]

S5. Relative distances of M_1, M_2, and M_3

Figure S6 shows the relative distances of M_1 and M_3, measured from M_2 obtained in the experiment in figure 3*a*. The blue, green, and red colors follow the same color scheme as in figures 1*c* and S3, and represent the corresponding trajectories. As expected, both distances (between M_3 and M_2, and between M_2 and M_1) undergo a reversal (reach an extreme point) during the process. Thus, M_1 reaches a furthest point and M_3 a closest point (at the same time). This plot also proves that the distance between M_2 and M_3 temporarily becomes shorter than between M_1 and M_2 when the system overcomes the activation potential.

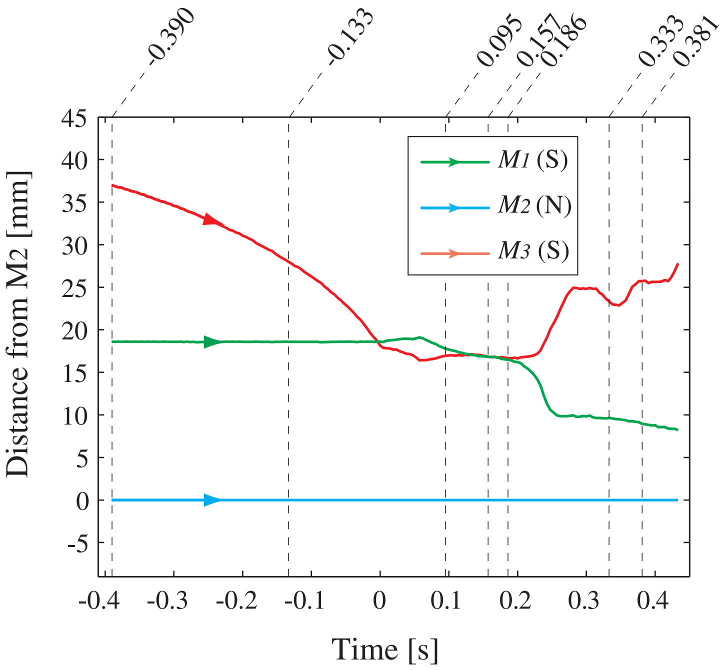

Figure S6: The relative distance from M_2 (blue color) to M_1 (green color) and M_3 (red color). The time stamps correspond to the snapshot frames in figure 3 *a*.

Movies

We provide 4 movies that display the typical behavior of (1) a conformation change invoked by $\mathcal{E}$ as shown in figure 3*a* (movie 1), (2) inhibition by $\mathcal{I}$ as shown in figure 3*b* (movie 2), (3) 3 trials of multiple unit combinations with $\mathcal{E}$s pre-docked on each $\mathcal{S}$ as shown in figure 4*a* (movie 3), and (4) 3 trials with $\mathcal{I}$s pre-docked on each $\mathcal{S}$ as shown in figure 4*b* (movie 4).

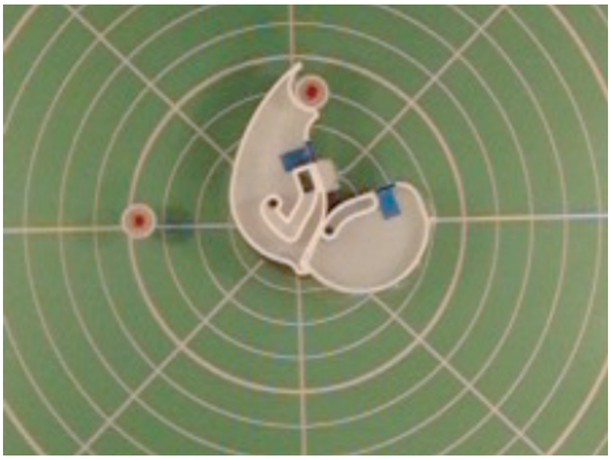

Figure S7: Conformation change of $\mathcal{S}$ triggered by a mobile $\mathcal{E}$, see figure 3*a*. The video is played in slow motion (1/14 $\times$ speed). movie 1

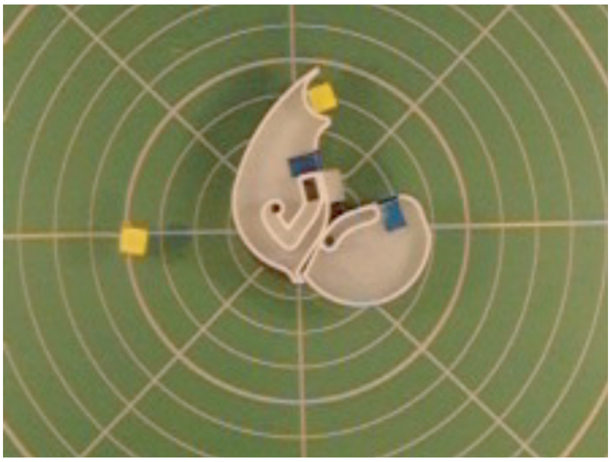

Figure S8: Inhibition of $\mathcal{S}$ by a mobile $\mathcal{I}$, see figure 3*b*. The video is played in slow motion (1/14 $\times$ speed). movie 2

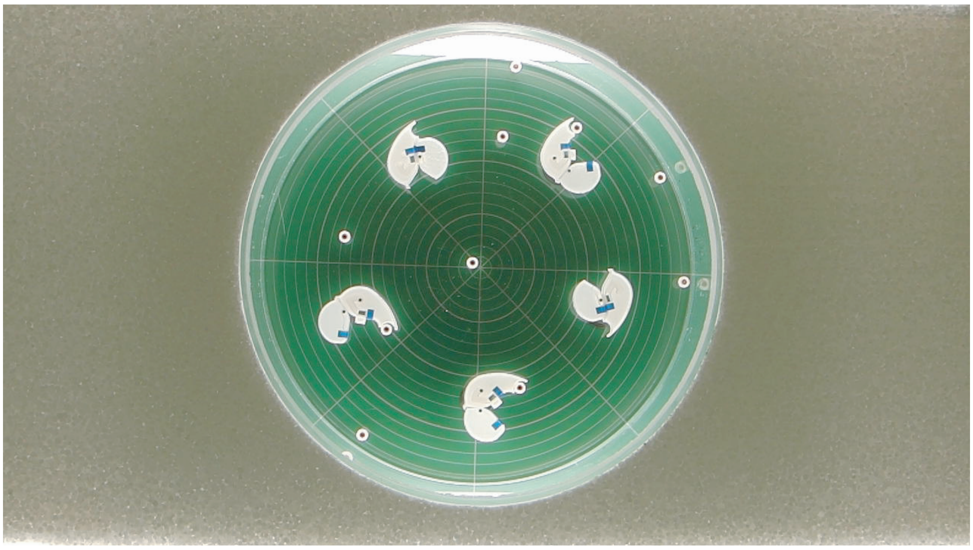

Figure S9: 3 representative trials showing autocatalysis with five units (with an $\mathcal{E}$ pre-docked on each $\mathcal{S}$). Snapshot of the first trial are shown in figure 4a. The video is played at $3\times$ speed. movie 3

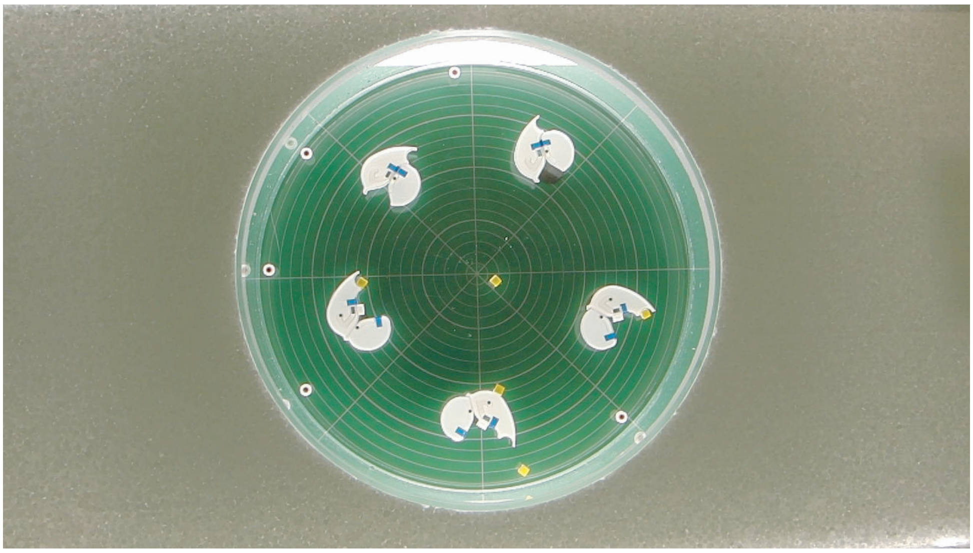

Figure S10: 3 representative trials showing inhibition with five units (with an $\mathcal{I}$ pre-docked on each $\mathcal{S}$). Snapshots of the first trial are shown in figure 4b. The video is played at $3\times$ speed. movie 4 (mov)
