# Supplementary material for: Mechanical catalysis on the centimetre scale
Source: J R Soc Interface. 2015 Mar 6;12(104):20141271. doi: 10.1098/rsif.2014.1271 (PMC4345491; doi:10.1098/rsif.2014.1271)
Supplement: Responses to referees [file rsif20141271supp2.pdf]

Responses to referees

Shuhei Miyashita, Christof Audretsch, Zoltàn Nagy, Rudolf M. Füchslin, and Rolf Pfeifer

We thank the referees for carefully reviewing our manuscript and providing comments and suggestions. We have modified the manuscript accordingly and responded to the points in the text below. In this response letter, the referees' comments are shown in red, whereas our responses are written in black. Text that has been modified or added to the original manuscript is set in *italics*, the *green* parts of which are the original version.

Referee(s)' Comments to Author:
Referee: 1

Comments to the Author
I believe the authors improved and paper and appreciate the clarifications they made. I also appreciate the opportunity to read the paper and understand some of its aspects that I had not understood but were already clear in the previous version. However, I still have some minor things that need to be more clearly written.

Mandatory Minor revision:

" Equation (2.1) holds strictly only for rij jj ≫ the magnet diameters, which is not always the case for us. However, it suffices to show the inter-magnet distance relationships."

It suffices for what? At what limit does this relationship breakdown and affects the design of your system and your ability to predict performance? A couple of short sentences are sufficient to address this comment.

Thank you for your comment. We have understood the importance and moved the following sentences from (former) Appendix B to Section 2.1 after equation (2.4) as:

> *Equations (2.1) - (2.4) hold strictly for rij ≫ the magnet diameters, and they gradually lose accuracy as rij becomes comparable to the diameters. However, since our model mostly needs to take the relative distances of magnets sets into account, they satisfy our requirements; in equation (2.3), the magnitudes of magnetic force strength are related to the relative distances, canceling out the inaccuracy of their values.*

Optional Revisions:

As far as I know there are still no standard report practices for reporting experiments done using devices printed in 3D printers. My believe is that such experiments should include the code used for printing the devices as well as the model of the printer. This is the only way in which reviewers and readers will be able to exactly reproduce and validate the reported experiments. As a matter of good practice I urge the authors to include this information as supplementary material as a SOP and Interface to define a code of good practice and minimum information description for such studies.

Thank you for your comment. We added a URL link to the uploaded CAD files in Section 3.4 "Experimental Setup" to make these files that we used in our experiments (SLDPRT and STL formats) downloadable. We added it as a footnote in the section as follows:

> *The CAD data of all the designed units can be downloaded as electronic material in STL file format at http://www.shuhei.net/.*

In our understanding, we think that the dependency on the capability of a 3D printer used in the reproduction of the designed geometry from a STL file is quite small, and the settings, e.g. density do not alter the outcomes. The experimental setup should be reproduced from the figures; just float printed units on water with magnets, and thus we believe providing detailed instructions may raise some

confusion. However, we will consider this matter and will provide appropriate supports for the readers through our website.

Referee: 2

Comments to the Author
The authors addressed most of review comments in the revised manuscript. I suggest to accept the manuscript. I have two minor comments:

1. In the conclusion: "The obtained model extends the conventional definition of catalysis to systems of alternative scales, realizing chemical reactions with hands-on artifacts, which can expand the concept of manufacturing."

I do not think any chemical reactions are demonstrated here. Chemical reactions refer to the process that affects the molecular structures. The authors presented an artificial system to catalyze the mechanical switch. Authors should revise this conclusion sentence with more precise definition.

Thank you for your comment. We have replaced the word *chemical* to “*mechanical*” and changed the sentence to:

> *The obtained model extends the conventional definition of catalysis to systems of alternative scales, realizing ~~chemical~~ “mechanical” reactions with hands-on artifacts, which can expand the concept of manufacturing.*

2. Appendix can be listed in the supplementary material, rather than occupy pages in the main text.

We have moved all the appendices to supplementary material. Thank you for your suggestion.
